# Paradoxical impact of sprawling intra-Urban Heat Islets: Reducing mean surface temperatures while enhancing local extremes

**DOI:** 10.1038/s41598-019-56091-w

**Published:** 2019-12-23

**Authors:** Anamika Shreevastava, Saiprasanth Bhalachandran, Gavan S. McGrath, Matthew Huber, P. Suresh C. Rao

**Affiliations:** 1 Lyles School of Civil Engineering, Purdue University, IN, USA; 2 Department of Earth, Atmospheric, and Planetary Sciences, Purdue University, IN, USA; 3 School of Earth and Environment, The University of Western Australia, Perth, WA Australia; 4 Ecosystem Science, Department of Biodiversity Conservation and Attractions, Kensington, WA Australia; 5 Present Address: Department of Earth System Science, Stanford University, CA, USA

**Keywords:** Atmospheric dynamics, Environmental impact, Natural hazards

## Abstract

Extreme heat is one of the deadliest health hazards that is projected to increase in intensity and persistence in the near future. Here, we tackle the problem of spatially heterogeneous heat distribution within urban areas. We develop a novel multi-scale metric of identifying emerging heat clusters at various percentile-based thermal thresholds and refer to them collectively as *intra-Urban Heat Islets*. Using remotely sensed Land Surface Temperatures, we first quantify the spatial organization of heat islets in cities at various degrees of sprawl and densification. We then condense the size, spacing, and intensity information about heterogeneous clusters into probability distributions that can be described using single scaling exponents (denoted by *β*, ${{\boldsymbol{\Lambda }}}_{{\boldsymbol{s}}{\boldsymbol{c}}{\boldsymbol{o}}{\boldsymbol{r}}{\boldsymbol{e}}}$, and *λ*, respectively). This allows for a seamless comparison of the heat islet characteristics across cities at varying spatial scales and improves on the traditional Surface Urban Heat Island (SUHI) Intensity as a bulk metric. Analysis of Heat Islet Size distributions demonstrates the emergence of two classes where the dense cities follow a Pareto distribution, and the sprawling cities show an exponential tempering of Pareto tail. This indicates a significantly reduced probability of encountering large heat islets for sprawling cities. In contrast, analysis of Heat Islet Intensity distributions indicates that while a sprawling configuration is favorable for reducing the mean SUHI Intensity of a city, for the same mean, it also results in higher local thermal extremes. This poses a paradox for urban designers in adopting expansion or densification as a growth trajectory to mitigate the UHI.

## Introduction

More than 50% of the world’s population currently resides in cities, and the number continues to increase rapidly with a projection that 70% of the global population will be urbanized by 2050^1^. Rapid urbanization trends are manifested in the expansion and densification of existing cities and the merging of small urban agglomerations to form megacities, particularly in South Asia and sub-Saharan Africa^2^. Among the numerous challenges that cities face, a particularly urgent problem due to climate change is that of extreme heat. Urban areas often raise the local temperatures relative to natural and rural surroundings leading to the phenomenon of the Urban Heat Island (UHI) effect. Synergistic interaction between UHIs and increasingly persistent heatwaves further exacerbates the extreme temperatures within cities^3,4^. Repercussions of extreme heat include thermal discomfort^5^, increased energy consumption^6^, and heat-related morbidity and mortality^7,8^.

The UHI is typically quantified as UHI Intensity, i.e., the difference between the air temperatures of a representative urban area (point measurement or spatial average) and rural area. However, such an estimate is inadequate to address the *intra-urban* spatial heterogeneity. Commendable efforts to collect spatially resolved thermal data such as the Basel Urban Boundary Layer Experiment (BUBBLE) campaign^9^ are rare and often limited to a single city. On the other hand, earth-monitoring satellites such as Landsat and Moderate Resolution Imaging Spectroradiometer (MODIS) enable consistent high spatial resolution characterization across multiple cities. As a result, the Surface UHI (SUHI) estimated using Land Surface Temperatures (LST) has emerged as an alternative approach that we adopt in this study^10,11^. While SUHI bears similarity in spatial patterns to UHI^12^, LST is more coupled with urban form and function, whereas air temperatures are subject to the boundary layer wind profiles as well. Therefore, a point-to-point correspondence can not be expected^13^.

The role of the spatial organization of urban form in reducing urban temperatures has been a topic of substantial research spanning a multitude of spatial scales. At the micro-scale, i.e., within the urban canyon, the surface temperatures are extremely sensitive to the geometrical details of immediate surroundings, such as street canyon geometry, sky-view factor, vegetative fraction, solar access and shading^14–16^. At the local scale, i.e., of the order of a few kilometers, consistent thermal patterns emerge due to locally homogeneous patches of urban form and function^17,18^. However, we do not have a clear understanding of the optimal urban form and function that minimize the urban heat locally as well as at a city-scale. For instance, studies investigating local scale impacts^19,20^ report that high-density urban development leads to higher local temperatures. In contrast, several others note that sprawling urban development may result in worse thermal conditions^21,22^. Despite these recent advances, a comprehensive framework for the characterization of intra-urban thermal heterogeneity for diverse city morphologies is still lacking. Towards that, we use a multi-scale framework wherein we treat the SUHI not as a single entity, but as a collection of heterogeneous clusters of heat within the city. We refer to these clusters as **intra-urban heat islets**. The objective of this study is to evaluate the impact of the spatial organization of these heat islets on their properties, such as size and intensity, and determine if there is a favorable spatial structure for reducing surface temperature extremes at intra-urban spatial scales.

Urban morphology, and as a result, LST, emerges via the processes of densification and expansion, albeit constrained by cultural, geographic, and economic factors^23,24^. Different degrees and combinations of these two processes result in a diversity of form and function. Dense urban growth occurs when there is increased in-fill construction within the existing high-density built-up area. Such a process is often driven by economic and socio-political factors that lead to the settlement of new urban regions close to the city center^25^. This is akin to the preferential attachment phenomenon observed in complex networks where a new node is more likely to attach at the “hub nodes” with the highest density of edges^26^. We hypothesize that the densification within urban areas results in hot regions getting hotter and larger, thereby resulting in power law, otherwise known as a Pareto, size distribution^27^ of heat islets. Urban expansion in the form of sprawl, on the other hand, occurs at the periphery of urban areas in the form of growing suburban regions. We hypothesize that this would lead to the emergence of heat islets that are spread more evenly throughout the city, often interspersed with local heat sinks. This can be detected in the size distribution as a fast decaying tail, often in the form of an exponential tempering^28^. Similar effects of urban expansion and densification are observed on the power law distributions prevelant in several urban infrastructure systems such as roads and sewage networks^29–31^. Note that we don’t refer to the spatial organization of urban assets such as buildings or impervious areas. Instead, we directly analyze the LST. We implement the framework for a set of 78 cities sampled globally. Using probabilistic models, we condense the size, spacing, and intensity information about heterogeneous clusters into distributions that can be described using single scaling exponents. This allows for a seamless comparison of the heat islet characteristics across cities that represent varying degrees of sprawl or densification. We then assess how the thermal spatial structure relates to the traditional lumped metric, SUHI Intensity. Lastly, we discuss implications for desirable thermal configurations for cities to minimize the area and intensity of the heat islets.

### Data acquisition and Clustering technique

**Figure 1:**
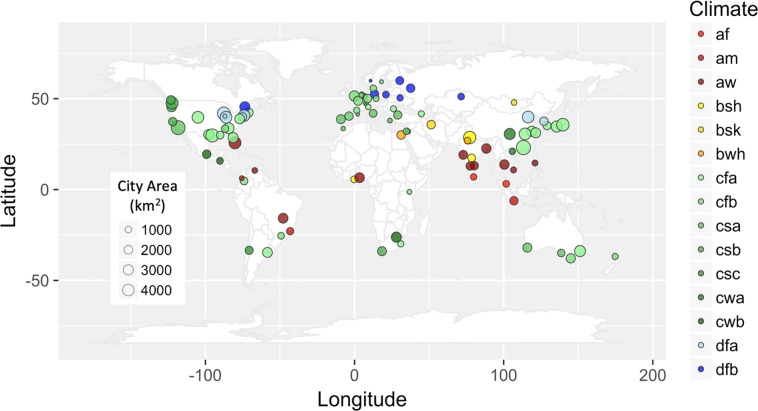
World map showing the locations of 78 cities considered in this study. The marker size is representative of the city size, and the colour represents their Koppen-Geiger climate classification^57^. Description of Koppen-Geiger climate types are given in Supplementary Table 1.

A set of 78 cities encompassing diverse climatological, geographical, and cultural backgrounds as well as different realizations of urban form and function were sampled (Fig. 1). The cities selected range from megalopolises such as Guangzhou, London, and New York City with a population of over 10 Million and metropolitan areas up to 3000 km^2^, to smaller cities such as Tbilisi, Bern, and Oslo that span less than 100 km^2^. As a globally standardized dataset of urban extent, the urban land use layer of MODIS Land Cover product was used. The exact definition of urban boundaries and city area plays a significant role in urban scaling laws^32^. Therefore, a buffer of 5 km in the rural regions was taken to account for the peri-urban settlements. However, as the heat islets occur well within the city boundaries, the results were found to be independent of the buffer width.

For each city, we selected a cloud-free Landsat image, and derived the LST in the geospatial computing environment of Google Earth Engine^33^ using the methodology described in Walawender *et al*.^34^. A complete list of Landsat imagery used for every city can be found in the Supplementary Dataset. A novel aspect of our methodology is the clustering technique used to characterize the LST. The LST maps are treated analogously to topography in Digital Elevation Models (DEM), where the temperatures substitute for elevation^35^. As the cities belong to diverse climatic backgrounds (and hence, different reference temperature)^36^, percentile-based thermal thresholds were chosen for identifying the relative hottest regions within the urban areas. The areas above each thermal threshold were identified, and the connected pixels were grouped into a cluster that we refer to as a **heat islet**. Supplementary Information provides code and text describing the methods in more detail.

## Size distribution of Heat Islets

In an exploration of *shapes* of heat islets, we found consistent self-similar, fractal topography across all cities^37^ (See Supplementary Fig. 1). Here, we focus on their *size* distribution by building on the scaling laws known for fractal surfaces. According to Korčák’s law^38^, the size distribution of clusters in a fractal topography is expected to follow the Pareto distribution, at the percolation threshold^39,40^. This is mathematically represented as: $N(a)\propto {a}^{-\beta }$ where *N* is the number of clusters of area, *a*, and the scaling exponent is *β*. Expressed as an exceedance probability we can write it as:

1 $$P(A\ge a)\propto {a}^{1-\beta },\,\forall \,a\ge {a}_{{\min }}$$

where, for a given area *a*, the probability of an islet having an area *A* larger than *a* is represented by *P*, the scaling exponent is represented by *β*, and the minimum area at or above which the power law is valid is represented as *a*_*min*_. We use Maximum Likelihood Estimation (MLE) to test for and fit the exceedance probability distributions^41^ (See Supplementary Text 3). This process is carried out for multiple thermal thresholds (50^th^, 60^th^, …, 90^th^ percentiles). We find that the estimated exponents ranged between 1.6 to 2.2 with a mean $\beta =1.88$. However, for the smaller cities (${A}_{city}\le 650\,{{\rm{km}}}^{2}$), the variability in exponents was much larger (Supplementary Fig. 2). One explanation for this is statistical, wherein for small cities, fewer islets obtained at 90 m resolution results in higher statistical fluctuations about the mean. As the number of islets increases with city size, larger sample sets are obtained, which results in a convergence of the scaling exponent towards the mean. However, from an urban growth perspective, this behavior is consistent with several other complex systems that operate within cities^31,42^. For smaller cities, the variability due to factors unrelated to city size, such as diversity of urban form, results in more detectable fluctuations. As cities grow in size, the spatial patterns converge due to self-organization^43^. We, therefore, excluded the smaller cities from any further analysis and proceeded with the remaining 49 cities where the internal thermal structure could be reliably quantified. For the larger cities, the distributions were well described by Eq. 1 with the same mean exponent and a narrow variability (std. dev. = 0.026).

**Figure 2:**
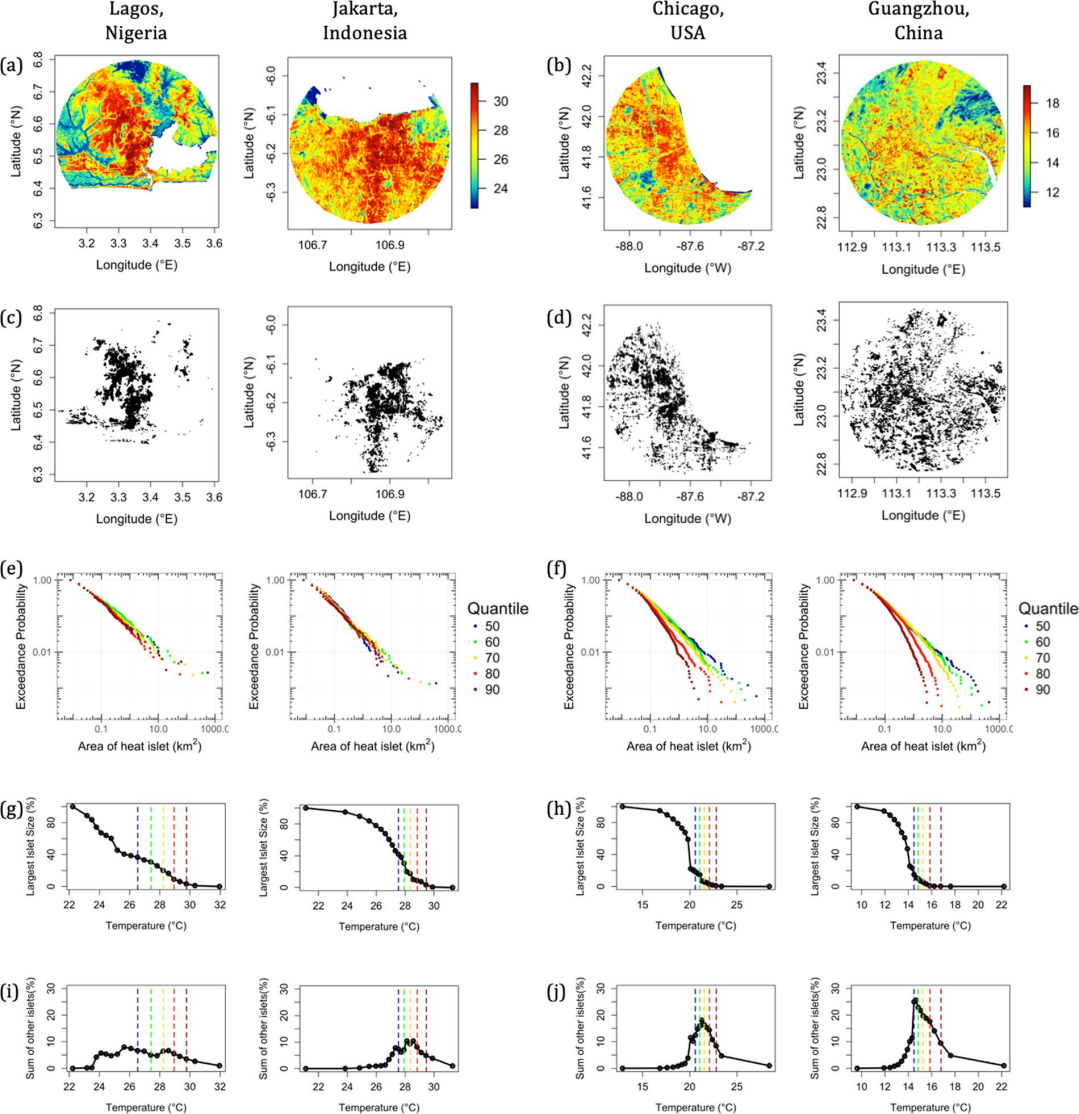
Two groups of cities emerge based on the size distributions of heat islets at incremental thermal thresholds. Two representative cities for each group - Jakarta, Indonesia, and Lagos, Nigeria for dense cities, and Chicago, USA, and Guangzhou, China for sprawling cities - are shown. (**a**,**b**) Land Surface Temperature map (in °C), (**c**,**d**) Heat islets that emerge at the 90^th^ percentile thermal threshold, (**e**,**f**) Exceedance probability plots for heat islets at several thermal thresholds (50^th^, …, 90^th^). Note the leftward shift in size distribution as the thresholds increase, especially the exponential tempering evident in sprawling cities, (**g**,**h**) Largest islet size, and (**i**,**j**) sum of remaining islets (as a % of total city area), as a function of thermal threshold. The vertical dashed colored lines mark the temperatures corresponding to the percentiles used in (**e**,**f**).

The Pareto size distribution is consistent at lower thresholds for all cities. However, the impact of a dense or sprawling spatial organization becomes apparent in how the exceedance probability distributions change as the threshold increases. The large metropolitan regions of Lagos and Jakarta are selected as representatives of dense cities, whereas Chicago and Guangzhou are chosen to represent sprawling cities (Fig. 2a,b). At 90^th^ percentile threshold, Lagos and Jakarta show a pronounced aggregation of heat islets indicative of the dominance of a dense urban center, whereas Chicago and Guangzhou are more dispersed (Fig. 2c,d). In agreement with our initial hypothesis, Lagos and Jakarta, display a Pareto heat islet size distribution across all the thresholds (Fig. 2e). However, for Chicago and Guangzhou, the heat islet size distributions deviate significantly from the Pareto in the form of an exponential tempering (Fig. 2f), such that their distributions more closely follow:

2 $$P(A\ge a)\propto {a}^{1-\beta }\cdot {e}^{-c\cdot a},\,\forall \,a\ge {a}_{{\min }}$$

where *c* represents the exponential tempering coefficient for each thermal threshold (See Supplementary Table 3 for the complete set).

Such behavior is explained by invoking percolation theory^44,45^. Percolation theory is a study of random clusters and their spatial connectivity at a given threshold. The coagulation of dispersed clusters into a contiguous component is referred to as percolation, and the largest cluster is identified as the percolating cluster. In fractal landscapes, the Pareto size distribution of clusters holds within a finite range (Percolation Transition Range) of thresholds, i.e., until the percolating cluster retains its identity. We computed the percolation transition range by identifying the inflection points in the size of the largest cluster as a function of temperature threshold (Fig. 2g,h). The range was then normalized using the minimum and maximum temperatures for each city such that the range is restricted to 0 and 1. We refer to this as the Normalized Percolation Range (NPR) (Supplementary Fig. 3). In the case of the aggregated cities (e.g., Jakarta and Lagos), as the temperature threshold is increased, the largest connected islet decreases in size gradually, and the resulting NPR is large (Fig. 2g). Conversely, in the case of sprawling cities (e.g., Chicago and Guangzhou), there is a much sharper decrease in the size of the percolating cluster (Fig. 2h) resulting in a narrow NPR (Fig. 2i,j). As the 90^th^ percentile thresholds in these cases fall outside the NPR (Fig. 2j), exponential tempering is observed.

From the perspective of the size distribution of heat islets alone, fewer and smaller heat islets are captured as the thermal threshold is increased. Therefore, an exponential tempering presents a reduced probability of encountering large heat islets of higher temperatures. This suggests that a sprawling spatial structure is favorable for reducing the size of extreme heat islets. Thus far, we have characterized the size distribution of these islets, not their spatial organization. We now introduce a metric to quantify and analyze the relationship between the spacing of the urban heat islets and the characteristics we observed in their size distributions.

## Quantifying Aggregated Versus Dispersed Heat Islets: Lacunarity

A built-up patch in a city acts as a source of increased sensible heat flux, as well as anthropogenic heat flux due to human activities such as air-conditioning. Likewise, the colder regions between the patches (also referred to as spacing in this work), especially large-scale urban parks and water bodies (such as New York’s Central Park or London’s Hyde Park), can help dissipate the excess heat generated. Therefore, characterizing this spacing between the urban patches is an essential step towards ameliorating heat stress^46^. Particularly, the impact of the relative sizes and strengths of such sources and sinks on the overall thermal landscape has been relatively understudied and requires further investigation. Since the present study focuses on the thermal landscape characterized by LST, we can directly quantify the spacing between the identified heat islets. Popular metrics such as root mean square distances work well for Gaussian systems, but for fractal landscapes, lacunarity is a better-suited metric of spatial structure^47^.

Lacunarity ($\Lambda$) is a scale-dependent measure of the aggregation of spaces between the heat islets^47,48^. A ‘gliding box’ algorithm for the calculation of $\Lambda$ as a function of box size (*r*), as described in Plotnick *et al*.^49^, was adopted here (Methods section). While the absolute values of $\Lambda$ offer little insight, the appropriate way to interpret lacunarity is in the context of the rate of change of $\Lambda$ as a function of *r*. If the value of $log(\Lambda (r))$ decreases at any scale (quantified with $log(r)$), the presence of spacing corresponding to that length scale is indicated. The two extremes of lacunarity curvature can be best conceptualized as a chessboard-type homogeneous distribution of small-scale spacing, and a single contiguous cluster. Essentially, the length scales corresponding to the steepest slopes should be interpreted as the dominant scale of spacing.

**Figure 3:**
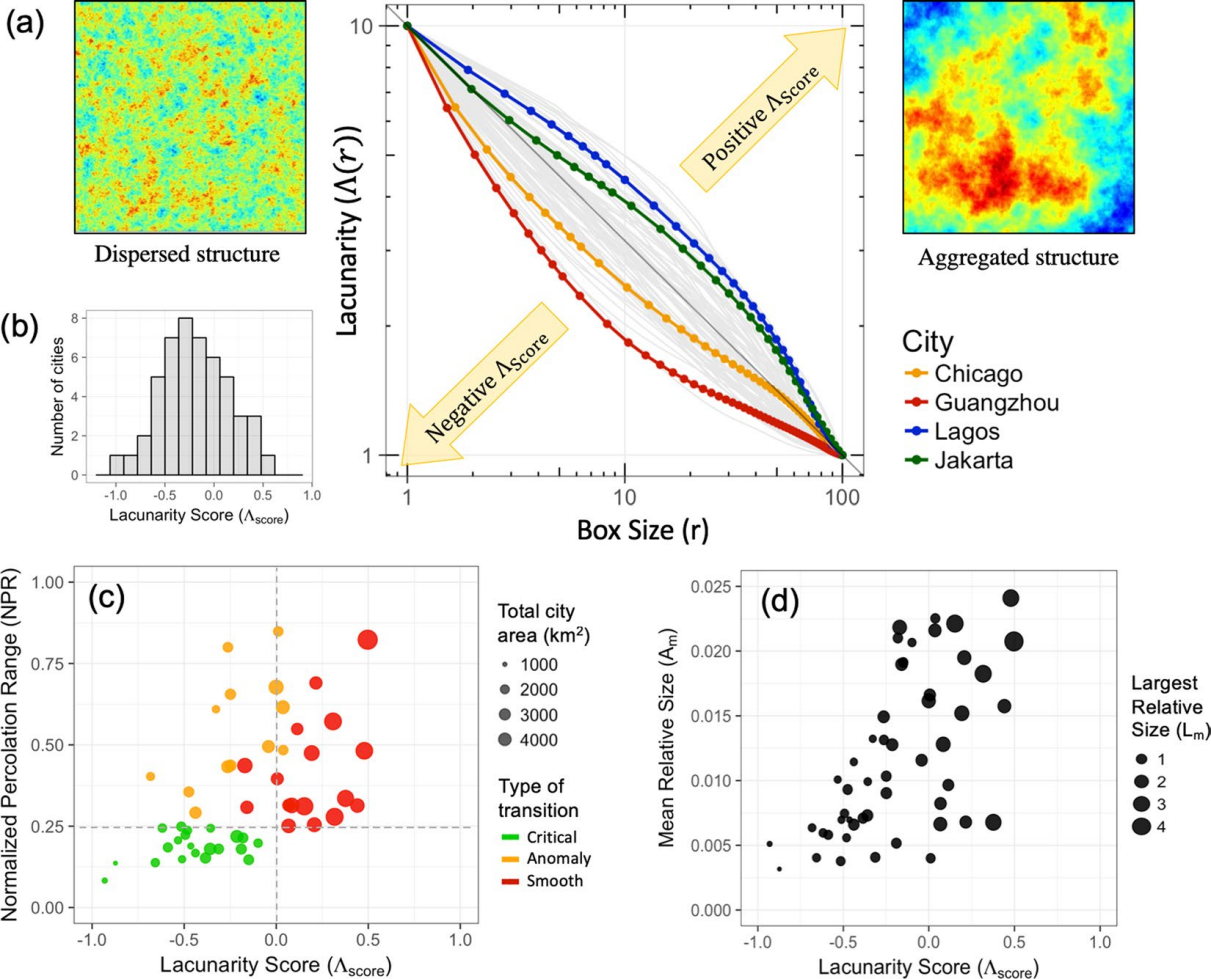
(**a**) Lacunarity curves of 49 cities (in grey) and the four archetype cities (in colour) shown on a *log*$(\Lambda )$ vs *log*(*r*) plot. The cities with a concave downwards shape in the upper side of the diagonal indicate larger and more aggregated gaps, whereas cities underneath the curve indicate a more uniform dispersed pattern of islets and smaller gaps. (**b**) Histogram of ${\Lambda }_{score}$ of 49 cities (mean = 0.04, s.d. = 0.38). (**c**) Scatter plot of percolation transition range and Lacunarity score. This figure illustrates the classification of cities into the 2 classes based on Lacunarity Score and the type of transition. (**d**) Scatter plot of Mean Relative Heat Islet Size (*A*_*M*_) versus ${\Lambda }_{score}$. Additionally, since the islet-size distribution is heavy tailed, in addition to the *A*_*M*_, the largest islet size (*A*_*L*_, as a percentage of the total city area) is indicated using the marker size. The *A*_*M*_ and *A*_*L*_ serve to illustrate the size distribution of the hottest islets occupying the ten percent of the city area.

As the differences in the spatial organization of heat islets are most apparent at higher temperature thresholds, here, we characterize the spatial structure obtained at the 90^th^ percentile of LST for all cities. In other words, the total *islet area* under consideration corresponds to the hottest 10% of the total city area. Lacunarity curves for the four representative cities investigated in the previous section are highlighted in Fig. 3. The cities that have an abundance of larger spacing between the islets lay above the diagonal. Conversely, a dispersed spatial structure of the heat islets manifests as smaller spacing and falls under the diagonal. We assign a single score (${\Lambda }_{score}$) to the convexity of the curves in Fig. 3a such that positive scores indicate larger spacing and vice-versa. This is achieved using the following empirical equation:

3 $$lo{g}_{10}(\Lambda (r))={(1-\frac{lo{g}_{10}(r)}{2})}^{{2}^{{\Lambda }_{score}}}$$

where constants 1 and 2 are used to fix the end points of the curve at $log(\Lambda (r))=1$ and $log(r)=2$, and the exponent, ${\Lambda }_{score}$ is scale-independent measure of the shape of the lacunarity curve (See Methods section). The 49 cities have ${\Lambda }_{score}$ ranging between −0.9 to 0.6, and distributed normally (Fig. 3b; See Supplementary Table 4).

Using ${\Lambda }_{score}$, we compare the relationship between the islet spacing and their NPR (and by extension, likely exponential tempering at higher thresholds). We find that the dense cities that are associated with an aggregated heat islet structure (positive ${\Lambda }_{score}$) display a larger NPR (≥0.25; Fig. 3c). Whereas, sprawling and disaggregated cities (negative ${\Lambda }_{score}$) have a smaller NPR (<0.25; Fig. 3c) and consequently an exponential tempering of the Pareto tail (Fig. 2f). An exception to this pattern are cities with a negative ${\Lambda }_{score}$ despite having an NPR ≥ 0.25 (shown in yellow in Fig. 3c). Upon examination, we found these to have a significant river flowing through them. Under such a scenario, the percolating heat cluster is divided structurally into two halves by a heat sink (the river), irrespective of the threshold (Supplementary Fig. 4). This results in a negative ${\Lambda }_{score}$ due to the spacing introduced by the river despite an aggregation of heat islets on either side of the river. Thus, Fig. 3c serves to quantitatively affirm the correlation between the spatial configuration of cities (dense versus sprawling) and the two classes of size distributions of the heat islets.

Note that for any given size distribution, the islets can be spatially arranged in several ways. To examine the variability in islet size and spacing of the various cities, we define two scale-independent metrics to characterize size: Mean (*A*_*M*_) and Largest (*A*_*L*_) Relative Heat Islet Sizes, calculated as a percentage of the total city area. First, we observe that there is a weak positive correlation (${R}^{2}=0.4$) between *A*_*M*_ and spacing of the heat-islets (Fig. 3d). This is expected because a positive ${\Lambda }_{score}$ as well as a high *A*_*M*_ corresponds to dense cities, and a negative ${\Lambda }_{score}$ and low *A*_*M*_ corresponds to sprawling cities. More noteworthy is the horizontal spread about the diagonal in Fig. 3d, which reflects the different spatial configurations (characterized by ${\Lambda }_{score}$) that are possible for any given size distribution. This spread may be explained by *A*_*L*_, which increases with ${\Lambda }_{score}$ (illustrated using marker size in Fig. 3d; Supplementary Fig. 5). In the bottom-left, both *A*_*M*_ and *A*_*L*_ are small. This is because negative ${\Lambda }_{score}$ corresponds to sprawling cities where large clusters were absent in the islet-size distribution (as inferred from the exponential tempering of Pareto). In the bottom-right, however, the dominance of the largest aggregated islet results in a positive ${\Lambda }_{score}$ despite a low *A*_*M*_ value. A schematic diagram drawn to represent each of the vertices of this plot is given as Supplementary Fig. 6. The phase plot of *A*_*M*_ and ${\Lambda }_{score}$ may be useful for city planners to gauge the current spatial structure of the thermal landscape of their cities and to determine mitigation strategies to achieve a more desirable state.

## Islet Intensity Distribution

**Figure 4:**
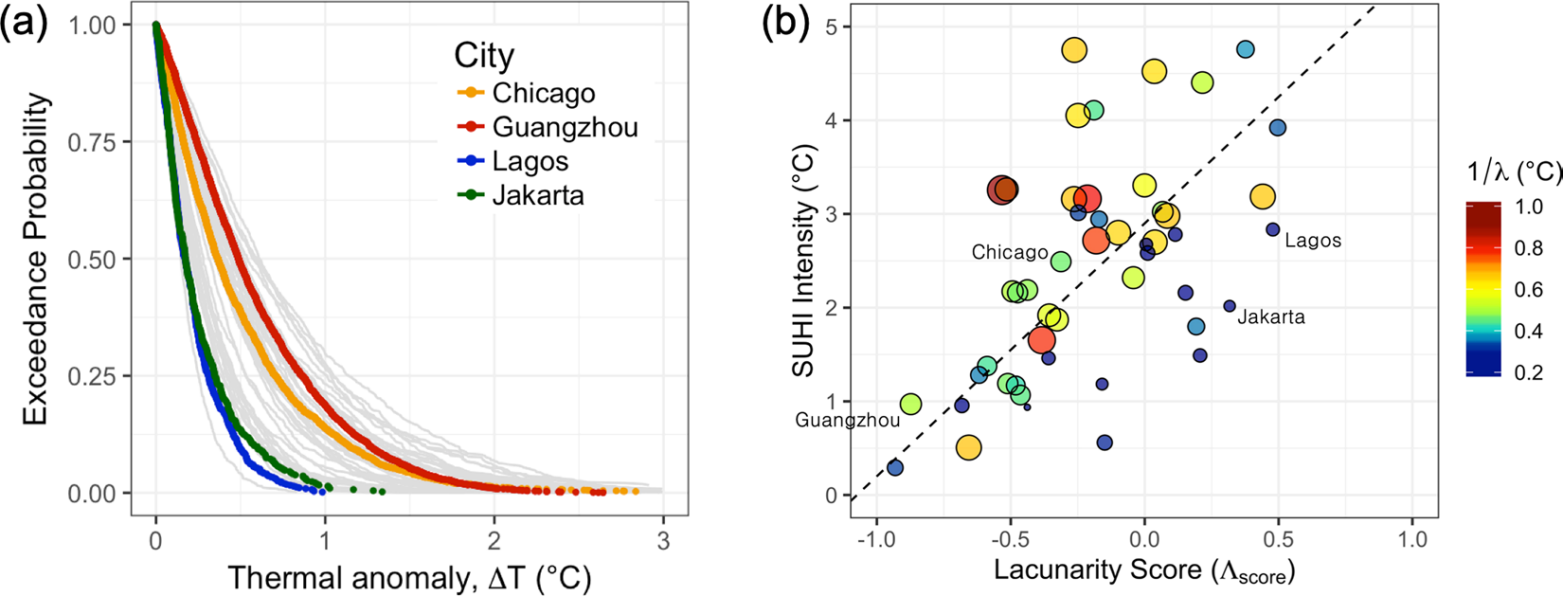
(**a**) Empirical pdf of Δ*T* for the four archetype cities shown on at their 90^th^ percentile thermal thresholds, respectively. The same for all 49 cities are shown in grey in the background. Each Δ*T* distribution was well described as an exponential distribution characterized by the parameter: *λ*. (**b**) A scatter plot of mean SUHI Intensity, defined as the difference between mean urban and rural temperatures versus Lacunarity Score (${\Lambda }_{score}$), is shown. A weak positive correlation (${R}^{2}=0.344$) is detected shown as dashed regression line. The color, as well as the size of the marker, indicates the inverse of rate parameter (*λ*) from Eq. 4, which is equal to the mean Heat Islet Intensity for each distribution. Increasing size indicates higher temperatures within the heat islets.

We now focus on the heterogeneity of heat contained within the heat islets. To address this, we first use the well-known indicator of excess heat in urban areas, the SUHI Intensity in the traditional sense, i.e., the difference between the mean urban and rural temperatures^50^ to evaluate the average excess heat within cities. We find that larger ${\Lambda }_{score}$ values (representative of aggregated heat islets) tend to be associated with higher SUHI Intensity (Fig. 4b). This suggests that sprawling cities, with a larger number of heat sinks to match the heat sources, are a better configuration for reducing the *overall* SUHI Intensity. This is in agreement with our findings based on the size distribution of extreme heat islets as well as prior research based on the discontiguity of urban patches derived from the National Land Cover Dataset (NLCD) for US cities^46^.

Traditional estimates of the UHI Intensity that simply use the difference between the **mean** temperatures over an urban area and the surrounding non-urban environment fail to address the intra-urban heterogeneity adequately. For a more comprehensive assessment of the thermal variability within cities, we introduce a novel *Heat Islet Intensity* distribution metric. First, we compute the excess heat (Δ*T*) for each islet as the difference between the mean *islet* temperature and the *threshold* temperature. We refer to this term as the *Islet Intensity*. We find that the mean and standard deviation of Δ*T* were equal (Supplementary Fig. 7) which, along with the shape of its distribution (Fig. 4a), were indicative that Δ*T* is exponentially distributed, i.e.:

4 $$P(\Delta T\ge x)\propto 1-{e}^{-\lambda x}$$

where the probability of an islet intensity, Δ*T*, exceeding a value *x* is represented by an exponential distribution characterized by *λ*. By extension, 1/*λ* is the mean islet intensity. Lower values of *λ* correspond to an increased probability of higher temperatures within the islets. Therefore, a single metric, *λ* can be used as an indicator to capture the intra-urban thermal variability across islets. This is represented as the color bar in Fig. 4b.

We find that while cities with a higher degree of sprawl have a lower mean temperature, for the same SUHI (Y-axis in Fig. 4b), cities with lower ${\Lambda }_{score}$ also experience a higher likelihood of encountering thermal extremes. For example, dense cities such as Lagos and Jakarta have a steeper exponential decaying rate than Chicago and Guangzhou, which drastically reduces the probability of local thermal extremes within their heat islets. While the probability of a heat islet being hotter than their 90^th^ percentile by 1 °C is almost zero for the first two, the likelihood increases to roughly 20% for the latter two (Fig. 4a). As the larger heat islets are often associated with the highest islet intensity as well, this can result in significantly large areas of extreme heat, especially for megacities like Guangzhou and Chicago. Such a finding reveals that while mean SUHI Intensity decreases with sprawling cities, for the same mean, they also experience higher local thermal extremes. This can have drastic heat-related health impacts if these local extremes are co-located with the vulnerable populations. As a result, it is essential to characterize the thermal heterogeneity within the cities, therefore, in addition to the mean SUHI Intensity, the islet intensity distribution can be adopted as a complementary metric.

## Summary and Conclusions

Cities grow through a combination of parallel and sequential episodes of expansion and densification. Depending on local preferences and constraints, neighborhoods are built with different spatial patterns, for example, from dense downtowns to sprawling suburbs. Factors like geographical topography, coastline, and intra-urban commuting time constrain expansion, whereas others such as local building laws limit densification. While there are several objective functions such as commuting travel time distribution, net carbon emissions, and socio-economical factors which urban form and functions are optimized for, here, we focus on the aspect of urban heat. More specifically, the spatial heterogeneity of extreme heat islets within urban areas. Towards that, we present a novel multi-scale framework that allows us to identify intra-urban heat islets for several thermal thresholds. Using this framework, we evaluate the impact of spatial organization, characterized by a Lacunarity-based score. ${\Lambda }_{score}$ is calibrated to lie between −1 and 1 corresponding to sprawling and dense configurations of heat islets, respectively. However, we do not observe a bi-modal distribution corresponding to the two distinct classes. Rather, ${\Lambda }_{score}$ was normally distributed around a mean value close to zero, indicating that most cities display a balance between sprawl and dense heat islet structure. Different combinations and degrees of expansion and densification yield a diverse array of spatial structures between the two extremes.

We then condense the size, spacing, and intensity information about heterogeneous clusters into probabilistic distributions that can be described using single scaling exponents. This allows for a seamless comparison of the intra-urban heat islet characteristics across cities at several spatial scales ranging from 90 meters (resolution of input data and corresponding to several urban blocks) up to a few thousand sq. Km (total area of large cities). We implement this framework for 78 globally representative cities to answer the following key questions. First, how many and how big are the emergent heat islets at multiple thermal thresholds? Second, how much hotter than the threshold are these heat islets? From the size distribution analysis, we demonstrate that islet sizes in dense cities follow and maintain a Pareto distribution across all temperature thresholds. In contrast, the sprawling cities show an exponential tempering of tails at higher thresholds. Such a tempering is favorable as it indicates a reduced emergence of large heat islets in the sprawling and dispersed spatial configurations. Additionally, a dispersed configuration results in lower *mean SUHI Intensity* over the city. On the other hand, from the islet intensity distribution analysis, we find that heat islet intensities (Δ*T*) can be modeled as exponential distributions, where dispersed configurations result in higher rate parameters (*λ)*. This implies a significantly higher probability of encountering extreme temperatures within the islets. In other words, while a sprawling configuration is favorable for reducing the mean temperature of a city, for the same mean SUHI intensity, it results in higher local thermal extremes. The implications of this from a design perspective are: (i) While designing sprawling cities, higher intra-urban variability should be expected. (ii) With changes in urban morphology, we can attempt to control the precise spatial location of the extreme temperatures such that the intense heat islets do not occur where most vulnerable populations reside, such as densely populated downtowns, or areas without access to air-conditioning such as urban slums.

Note that LSTs are sensitive to seasonal and diurnal variabilities, as well as observational limitations such as time of Landsat overpass. As our methodology simply reflects the organization of LSTs, the metrics are sensitive to the variability in LST observations by extension (Supplementary Fig. 8). Furthermore, while the spatial characterization of temperature is informative for urban heat assessments, it does not inform the overall risk to the concerned population. Risk is a combination of hazard (i.e., extreme heat-stress in this case, a combination of air temperature and humidity^51^), the time period of exposure to heat stress, and vulnerability. Prior research shows that in the absence of hydrological processes, the changes in radiative flux, determined by LST, contribute more to the near-surface air temperature changes than the turbulent heat flux^52^. The developed methodology can be extended to air temperature and relative humidity (and by extension, heat stress) datasets as well for improved characterization of heat exposure. Similarly, vulnerability assessments will require further input variables describing demographic factors such as old age, low educational attainment, high poverty levels, poor health, and lack of air conditioning^8,53,54^. An investigation of the spatio-temporal dynamics of risk to extreme heat is beyond the scope of the present study, but it certainly warrants further research.

Lastly, our analysis here is limited to the structural heterogeneity of heat sources and sinks, wherein the sizes of heat islets are indicative of the strength of the sources, and the length-scale of spacing is indicative of the sink strengths. However, as heat capacity, as well as thermal conductivities, are highly heterogeneous in urban areas, consideration of the *functional* heterogeneity will require that we incorporate these variables as well. This could be made possible using models such as Weather Research Forecast (WRF)^55,56^. In such a scenario, instead of LST, heat fluxes can be treated as DEM for such an analysis. It may also then be beneficial to study the spatial correlation between source strength and sink strength to evaluate thermal dissipation. To conclude, our work presents the first steps toward a multi-scale characterization of the complex intra-urban thermal landscape, and we hope that it opens new vistas for future investigations.

## Methods

### Study area and data sources

Land surface temperature (LST) data were derived using a Single Channel Algorithm as detailed in^34^ from Landsat 8 at a resolution of 90 m. The geospatial analysis environment of Google Earth Engine (GEE) was used to collect data for representative cloud-free days^33^. R was used for all subsequent geospatial analyses. See Supplementary Text S1 and S2 for algorithms and Table S1 for a list of cities and information on Landsat scenes used. For coastal cities, the Large Scale International Boundary (LSIB) dataset provided by the United States Office of the Geographer was used to crop out the oceans and delineate urban boundaries within the GEE environment. The urban areas were estimated using MODIS’s Land Cover Type dataset - MCD12Q1.

### Statistical modeling of size and intensity distributions

For fitting probability distribution functions (pdfs) to cluster size and intensity distributions, a combination of maximum-likelihood estimation (MLE) with goodness-of-fit tests based on the Kolmogorov-Smirnov (KS) statistic and likelihood ratios were used^41^. See Supplementary Text S3 for details and Table S2 for results.

### Lacunarity

First, the landscape was sliced at a thermal threshold, and an islets map was obtained. For each box size ($1 < r < {A}_{city}$), the number of occupied pixels (islets) was measured. The number of occupied sites was referred to as the box mass. The box was then moved one column to the right, and the box mass was again counted. This process was repeated over all rows and columns, producing a frequency distribution of the box masses. The number of boxes of size r containing S occupied sites were designated by n(S,r) and the total number of boxes of size r by N(r). This frequency distribution was converted into a probability distribution: $Q(S,r)=\frac{n(S,r)}{N(r)}$. Lacunarity is a measure of variability in the calculated occupancy for each box size.

5 $$\Lambda (r)=\frac{Variance[Q(S,r)]}{Mean{[Q(S,r)]}^{2}}+1$$

For all cities, the Lacunarity score was calculated only for the 90^th^ percentile thermal threshold. As a result, 90% of the total area in all cases comprised of spaces, and the $\Lambda (r)$ value for box size = 1 was the same for all cities. The largest box size taken under consideration was normalized from 0 to 100 to account for the variable sizes of cities. Note that the curvature of the Lacunarity curve was unaffected by these transformations.

## Supplementary information

Supplementary Information

Supplementary Information 2
