# Supplementary material for: Paradoxical impact of sprawling intra-Urban Heat Islets: Reducing mean surface temperatures while enhancing local extremes
Source: Sci Rep. 2019 Dec 23;9:19681. doi: 10.1038/s41598-019-56091-w (PMC6928021; doi:10.1038/s41598-019-56091-w)
Supplement: Supplementary file 1 — Supplementary Information [file 41598_2019_56091_MOESM1_ESM.pdf]

Supplementary Information for

Paradoxical impact of sprawling intra-Urban Heat Islets: Reducing mean surface temperatures while enhancing local extremes

Anamika Shreevastava, Saiprasanth Bhalachandran, Gavan McGrath,

Matthew Huber, and P. Suresh C. Rao

Contents

1 Supplementary Text 1: Algorithm in Google Earth Engine used to retrieve relevant Landsat images for a selected city 2

2 Supplementary Text 2: Algorithm used on Landsat bands to compute Land Surface Temperature (LST) 4

3 Supplementary Text 3: Fitting Power law distributions 6

4 Supplementary Figure 1: Fractal Dimension 7

5 Supplementary Figure 2: Slope of Size distributions vs Area 7

6 Supplementary Figure 3: Normalized Percolation Range 8

7 Supplementary Figure 4: Cities with rivers 9

8 Supplementary Figure 5: Correlation coefficients of size vs spacing 10

9 Supplementary Figure 6: Schematic diagrams for Size vs Spacing 10

10 Supplementary Figure 7: Exponential distribution of thermal anomalies 11

11 Supplementary Figure 8: Lacunarity Test 12

12 Supplementary Table 1: Koppen Geiger Climate Classification 13

13 Supplementary Table 2: List of selected cities, location, Landsat scene information, and background Koppen Geiger climate information used. 13

14 Supplementary Table 3: Results of Log-likelihood statistics for testing power law tails and checking alternate distributions as discussed in Text S3 13

15 Supplementary Table 4: Metadata on the final results obtained for final 49 cities. 13

Supplementary Text 1: Algorithm In Google Earth Engine Used To Retrieve Relevant Landsat Images For A Selected City

Google Earth Engine (GEE) is the new planetary scale Geospatial analysis platform. It combines a multi-petabyte catalog of satellite imagery and geospatial datasets obtained from satellite such as Landsat and MODIS, and Google's massive computational capabilities. GEE (`https://code.earthengine.google.com/`) environment was used to obtain relevant Landsat and MODIS images of the 100 selected cities using the following code. Similar algorithm was used with MODIS data as an input to obtain land use information from MODIS.

```
Imports (4 entries)
  var Landsat8: ImageCollection "USGS Landsat 8 Collection 1 Tier 1 …
  var Coast: Table "LSIB: Large Scale International Boundary Polygon…
  var WorldPop: ImageCollection "WorldPop Project Population Data: E…
  var US_counties: Fusion Table "Merge of County Geometry and Census…


// Step 1: Variables to be changed: Find the location by clicking on
// inspector and then clicking on the location from the map.

var lat=    121.4539;
var lon=    31.2063;
var Radius=   25000;

var point = ee.Geometry.Point(lon,lat); // Location for the city

// Step 2: Select the time period of interest.

var start = ee.Date('2013-07-13');
var finish = ee.Date('2018-12-01');

// Step 3: Create a sub-collection of landsat images selected thereby
// Optional filters: Landsat Path and Row (if known).
// Then sort the collection by Cloud cover.

var regionCollection = Landsat8
  .filterBounds(point)
  .filterDate(start,finish)
// .filter(ee.Filter.eq('WRS_PATH', 175))
// .filter(ee.Filter.eq('WRS_ROW', 84))
// .sort('CLOUD_COVER', true);

var timeCollection = regionCollection
  .filter(ee.Filter.calendarRange(5,7,'month'))
  .sort('CLOUD_COVER', true);

print(timeCollection);
```

```

// Note that the images in the collection are a List stored in the
// ‘features’ property of the filteredCollection. The ID of any image
// in the collection can be copied into the Image constructor as above.
// Alternatively, get the first image (lowest cloud cover, as sorted):

var image = ee.Image(timeCollection.first());

// Define visualization parameters to see the selection on map
var vizParams = {bands: ['B4', 'B3', 'B2'], min: 5000, max: 14000};

// Center the map on the image and display.
Map.setCenter(lon,lat, 10);
Map.addLayer(image, vizParams, 'true color');

// Step 4: Clip the area using a buffer radius
var roi = ee.Geometry.Point([lon,lat]).buffer(Radius);
var City = image.clip(roi);

// Step 5: Crop out the oceans
var City = City.clip(Coast);
Map.addLayer(City, vizParams, 'true color');

// Step 6: Print out relevant metadata
print(City);
var Solar = City.get('SUN_ELEVATION');
print('SUN_ELEVATION: ', Solar); // ee.Number
var Clouds = City.get('CLOUD_COVER');
print('CLOUD_COVER: ', Clouds); // ee.Number
var date = ee.Date(City.get('system:time_start'));
print('Timestamp: ', date); // ee.Date

// Step 7: Save the Image
Export.image.toDrive({
  image: City,
  description: 'imageToDriveExample',
  scale: 30,
  region: roi,
});


// End
```

Supplementary Text 2: Algorithm Used On Landsat Bands To Compute Land Surface Temperature (LST)

Until recently, studies of urban effects on meteorology and climate have been conducted for isolated locations and with in-situ measurements. With the advent of high-resolution Earth-monitoring satellites, it has become possible to study these effects both remotely and on continental or global scales[1]. Here, we use LST derived from Landsat thermal bands for the analysis. While the native resolution of TIRS (bands 10 and 11) is 100 m, they are sampled to match the other bands at 30 m in the Landsat composite product [1]. In order to avoid any error that might have been introduced due to the downscaling of thermal band datasets, we opted to aggregate the resolution to 90m (which is closer to the native TIRS resolution). The derived temperatures are surface temperatures of the emitting materials. Given the abundance of surface types in the urban environment, it can cause surface temperatures to exhibit a much greater spatial variation than the concurrent air temperatures. Therefore, outliers outside 99.9 percentile were smoothed out by assigning them the average value of their neighborhood pixels.

The algorithm employed for the computation of LST here doesn't account of atmospheric correction. However, a systematic error throughout the Landsat scene is acceptable in this particular study because the absolute temperatures are not of interest, but the relative temperatures matter. The algorithm used as well as the R-code written for LST is outlined below.

Step 1: TOA radiance

$$L_\lambda = M_L \cdot Q_{cal} + A_L \tag{1}$$

where,
L_λ = TOA spectral radiance ($W/m^2 * srad * \mu m$)
M_L = Band-specific multiplicative rescaling factor from the metadata
(RADIANCE_MULT_BAND_x, where x is the band number)
A_L = Band-specific additive rescaling factor from the metadata
(RADIANCE_ADD_BAND_x, where x is the band number)
Q_{cal} = Quantized and calibrated standard product pixel values (DN)

Step 2: TOA Brightness Temperature

$$T = \frac{K_2}{ln(\frac{K_1}{L_\lambda} + 1)} \tag{2}$$

where,
T = At-satellite brightness temperature (K)
L_λ = TOA spectral radiance ($W/m^2 * srad * \mu m$)
K_1 = Band-specific thermal conversion constant from the metadata
(K1_CONSTANT_BAND_x, where x is the thermal band number)
K_2 = Band-specific thermal conversion constant from the metadata
(K2_CONSTANT_BAND_x, where x is the thermal band number)

The band specific values were obtained from the metadata file. These equations are used for both band 10 and 11, to obtain the temperatures. However, to obtain the actual ground

[1] Variable GRID_CELL_SIZE_THERMAL listed on page 27 of `https://prd-wret.s3-us-west-2.amazonaws.com/assets/palladium/production/atoms/files/LSDS-809-Landsat8-Level1DFCB-v11.pdf`

surface temperature, the emissivity needs to be calculated. The codes implemented in R here were derived and modified from ArcGIS toolbox[2].

Step 3: Proportion of vegetation (P_v) and Emmissivity (e) is estimated from NDVI to estimate actual LST:

$$P_v = \frac{NDVI - NDVI_{min}}{(NDVI_{max} - NDVI_{min})^2} \tag{3}$$

$$e = 0.004 * P_v + 0.986 \tag{4}$$

$$LST = \frac{T}{1 + w * \frac{T}{\rho} * ln(e)} \tag{5}$$

where,
T = At satellite brightness temperature (K) as per equation 4
w = Wavelength of emitted radiation (11.5 μm)
$\rho = h \times \frac{c}{\sigma} = 14380\ \mu mK$
(σ = Boltzmann constant = $1.38 \times 10^{-23}\ \frac{J}{K}$, h = Planck's constant = $6.626 \times 10^{-34}\ Js$, c = velocity of light = $2.998 \times 10^{8}\ \frac{m}{s}$)
e = emissivity as per equation 4

```
Landsat_to_LST <- function(input_landsat,Solar_elev){

  tic("Landsat to LST")

  Band4 <- raster(input_landsat,band=4)
  Band5 <- raster(input_landsat,band=5)
  Band10 <- raster(input_landsat,band=10)
  Band11 <- raster(input_landsat,band=11)

  DN_Red <- (0.00002*Band4 - 0.1)/sin(Solar_elev*(pi/180))
  DN_NIR <- (0.00002*Band5 - 0.1)/sin(Solar_elev*(pi/180))
  NDVI <- (DN_NIR-DN_Red)/(DN_NIR+DN_Red)
  NDVI_min <- minValue(NDVI)
  NDVI_max <- maxValue(NDVI)
  Emissivity <- 0.004*((NDVI-NDVI_min)/(NDVI_max-NDVI_min))^2 + 0.986
  Rad10<- 0.0003342*Band10 + 0.1
  SatTemp10<- 1321.0789/log((774.8853/Rad10)+1)
  LST10 <- SatTemp10/(1 + 10.8*(SatTemp10/14380)*log(Emissivity)) - 273.15

  Rad11<- 0.0003342*Band11 +0.1
  SatTemp11<- 1321.0789/log((774.8853/Rad11)+1)
  LST11 <- SatTemp11/(1 + 10.8*(SatTemp11/14380)*log(Emissivity)) - 273.15

  LST <- mean(LST10,LST11)
  toc()
  return(LST)

}
```

(Source: `https://landsat.usgs.gov/using-usgs-landsat-8-product`)

Supplementary Text 3: Fitting Power Law Distributions

For fitting probability distributions to the cluster size distribution, a combination of maximum-likelihood fitting methods with goodness-of-fit tests based on the Kolmogorov-Smirnov (KS) statistic and likelihood ratios were used [3]. A step-by-step methodology as summarized in Box 1 of the paper (as outlined below) was followed with the help of R-code provided by Laurent Dubroca and Cosma Shalizi on Clauset's website: `http://tuvalu.santafe.edu/~aaronc/powerlaws/`. Following their R-code for the analysis of power-law distributions the steps are as follows:

1. Estimate the parameters x_{min} and α of the power-law model.
2. Calculate the goodness-of-fit between the data and the power law. If the resulting $p - value \geq 0.1$, the power law is a plausible hypothesis for the data, otherwise it is rejected.
3. Compare the power law with alternative hypotheses via a likelihood ratio test. For each alternative, if the calculated likelihood ratio is significantly different from zero, then its sign indicates whether or not the alternative is favored over the power-law model.

The data was tested for a power law tail fit and compared against 4 other competing distributions - Exponential, Lognormal, Stretched Exponential (Weibull), and Power law with exponential rate of tempering. The equations are given below (adapted from Clauset, et al. 2009) [3].

	Name	Distribution $p(x) = Cf(x)$ $f(x)$	C
Continuous	Power law	$x^{-\alpha}$	$(\alpha-1)x_{\min}^{\alpha-1}$
	Power law with cutoff	$x^{-\alpha}\mathrm{e}^{-\lambda x}$	$\frac{\lambda^{1-\alpha}}{\Gamma(1-\alpha,\lambda x_{\min})}$
	Exponential	$\mathrm{e}^{-\lambda x}$	$\lambda \mathrm{e}^{\lambda x_{\min}}$
	Stretched exponential	$x^{\beta-1}\mathrm{e}^{-\lambda x^{\beta}}$	$\beta\lambda \mathrm{e}^{\lambda x_{\min}^{\beta}}$
	Log-normal	$\frac{1}{x}\exp\left[-\frac{(\ln x-\mu)^2}{2\sigma^2}\right]$	$\sqrt{\frac{2}{\pi\sigma^2}}\left[\mathrm{erfc}\left(\frac{\ln x_{\min}-\mu}{\sqrt{2}\sigma}\right)\right]^{-1}$

The basic idea behind the likelihood ratio test is to compute the likelihood of the data under two competing distributions. The one with the higher likelihood is then the better fit. Alternatively, one can calculate the ratio of the two likelihoods, or equivalently the logarithm R of the ratio, which is positive or negative depending on which distribution is better, or zero in the event of a tie. Furthermore, the p-value for the Log-likelihood Ratio is checked and an outcome is selected only if the p-value < 0.1 (For a 90% confidence).

The cluster size distributions for all cities were tested at several thermal thresholds based on the following percentiles: 50th, 60th, 70th, 80th, and 90th. All of the distributions were found to qualify as a power law tail (with a p-value of 0.1, i.e. 90% confidence) for lower percentile thresholds. The lower cut-off for power law was found to be under 500 m for most cities (95% CI one-sided), this roughly corresponds to the size of an urban block implying that the scaling doesn't extend to the length scales smaller than an urban block. At 90th percentile threshold, we find that 25 of the 78 cities were described as a power-law with exponential tempering: $P(A > a) \propto a^{1-\beta}e^{-c \cdot a}$. However, none of them have likelihoods suggesting a Weibull, exponential, or lognormal describe the data better. The table with complete results is attached as separate excel sheet.

Supplementary Figure 1: Fractal Dimension

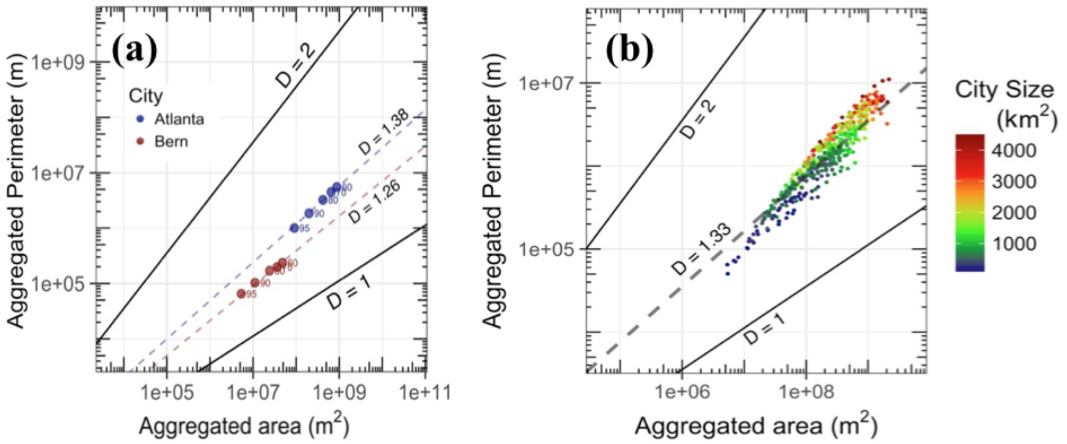

Figure 1: This figure was adapted from ref. [4]. (a) Aggregated perimeters versus aggregated areas of two cities, Bern and Atlanta, demonstrating the same ratio of $log(Area)$ and $log(Perimeter)$ for thresholds at 60, 70, 80, and 90 percentiles. The fractal dimensions of the perimeter of a circle ($D = 1$) and a space-filling plane ($D = 2$) are plotted to show the physical bounds. (b) Same as (a) for all cities shown with colour representing city-size. The grey, dashed line indicates the mean fractal dimension, $D = 1.33$.

Supplementary Figure 2: Slope Of Size Distributions Vs Area

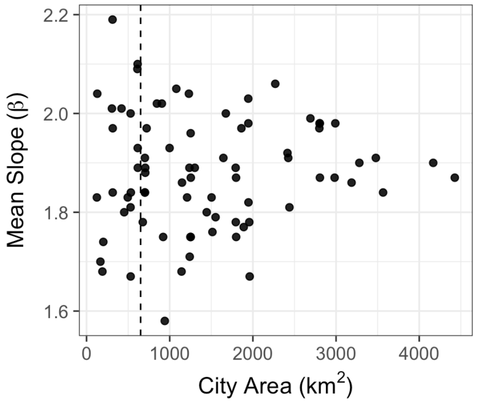

Figure 2: Scatter plots of City Area and mean slope of size distribution (β) for all cities. Vertical dashed line is corresponding to $A_{city} = 650\ km^2$. This figure serves to illustrate that the mean slope for cities below 650 km^2 also have a mean slope of 1.88 like the larger cities but with higher variability. The smaller cities were excluded from subsequent analysis because the number of heat islets obtained weren't enough to fit any statistically significant size distribution.

Supplementary Figure 3: Normalized Percolation Range

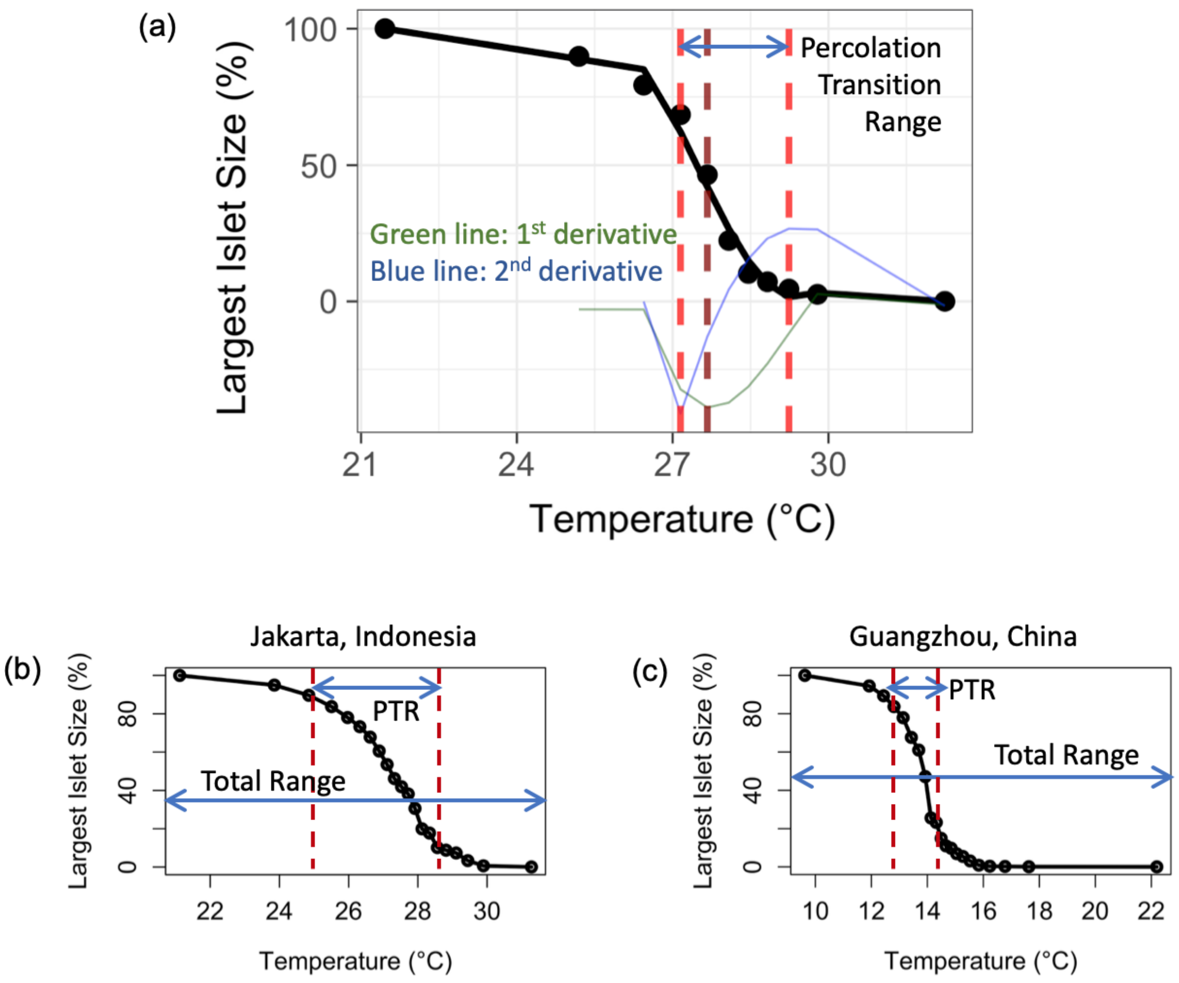

Figure 3: This figures serves to diagrammatically illustrate the Normalized Percolation Range (NPR). (a) Largest cluster size (A_L) as a function of temperature (T) was plotted. d^2A_L/dT^2 (shown in blue) at each thermal threshold was computed to find the inflection points. The range where rapid decrease in the largest cluster size takes place is referred to as the Percolation Transition Range (PTR). PTR is then divided by the total range to obtain the Normalized Percolation Range (NPR) for each city. Further examples of the same are shown as (b) Jakarta, and (c) Guangzhou.

Supplementary Figure 4: Cities With Rivers

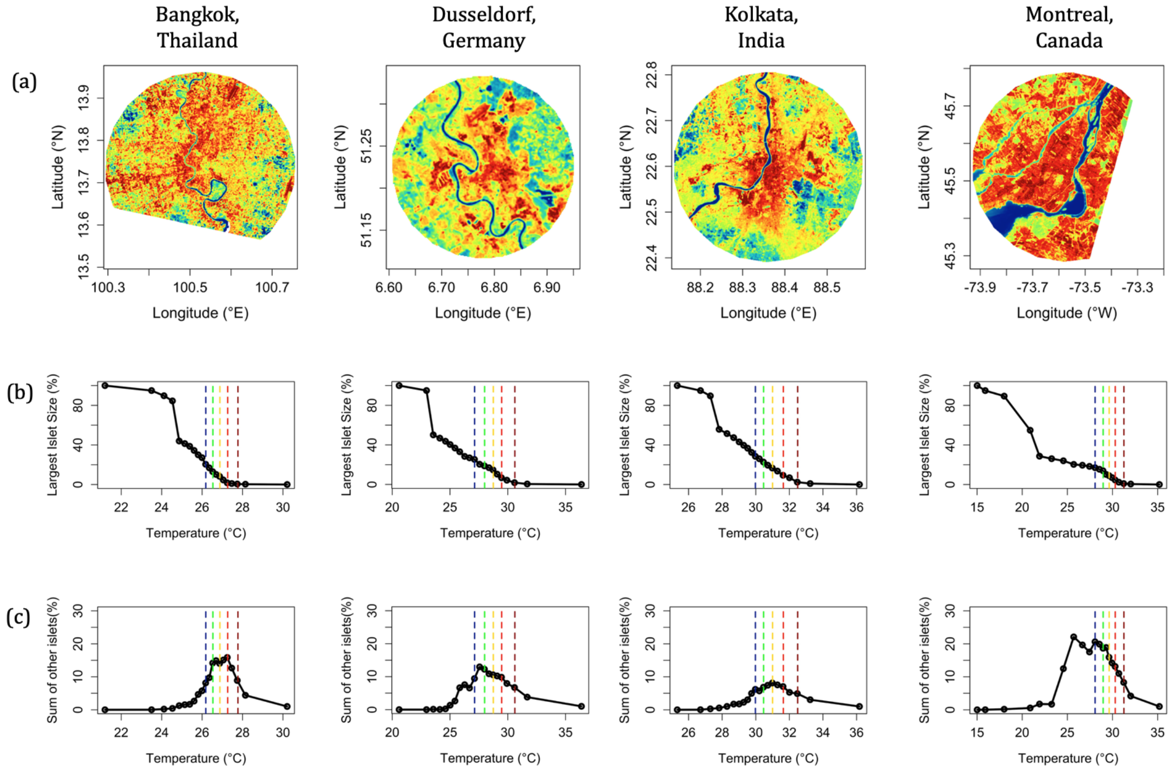

Figure 4: Some examples of anomalous cities with a river flowing between them resulting in a negative Λ_{score} and large Normalized Percolation Range (Figure 3b) are shown here. (a) Land Surface Temperature maps for Bangkok, Dusseldorf, Kolkata, and Montreal. (b) Largest cluster size (A_L) as a function of thermal threshold shows the sharp decrease in cluster size at lower thresholds which is **not** corresponding to a rise in size of other clusters. This is because the largest cluster merely breaks into two in these cases due to a central river (at low temperature. As a result, these are not classified as a critical transition as per percolation theory.

Supplementary Figure 5: Correlation Coefficients Of Size Vs Spacing

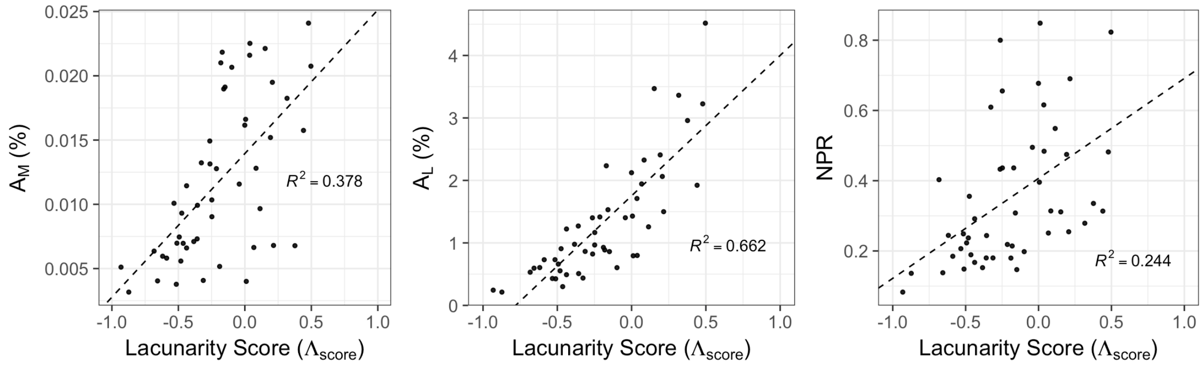

Figure 5: Scatter plots of Lacunarity Score (Λ_{Score}) and (a) Mean heat islet area (A_M), (b) Largest heat islet area (A_L), and (c) Normalized Percolation Range (NPR) and their correlation coefficients respectively.

Supplementary Figure 6: Schematic Diagrams For Size Vs Spacing

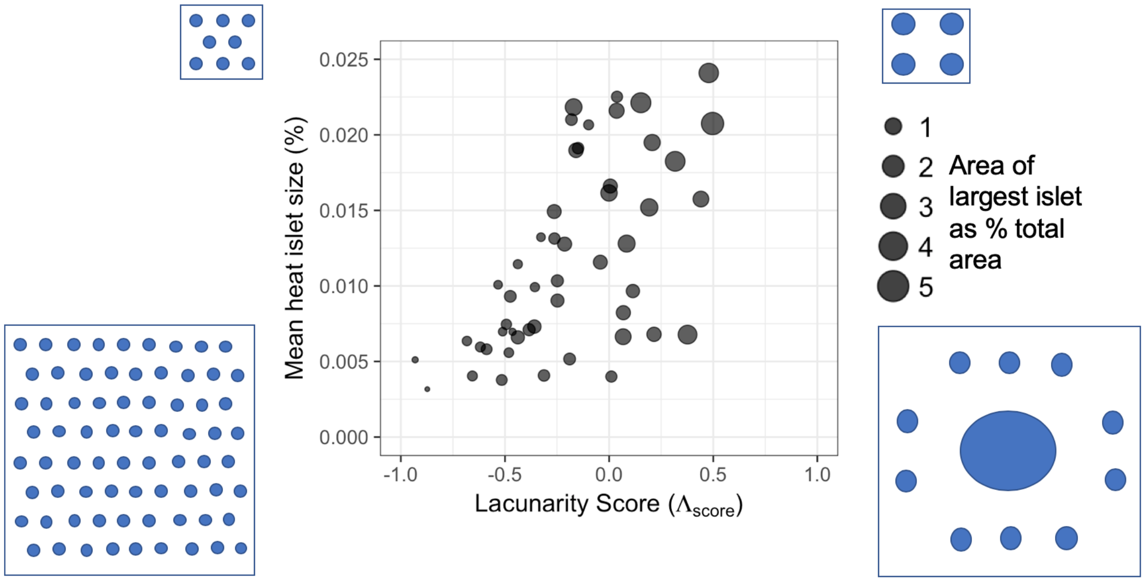

Figure 6: Scatter plot of Mean Relative Heat Islet Size (A_M) versus Λ_{score}. Additionally, since the islet-size distribution is heavy tailed, in addition to the A_M, the largest islet size (as a percentage of the total city area) is indicated using the marker size. The A_M and the largest-heat islet size (A_L) serve to illustrate the size distribution of the hottest islets occupying the ten percent of the city area. On the sides, corresponding to each quadrant of the phase space, schematic diagrams of spatial structure of heat islets are shown to better explain the various spatial configurations that are possible for cities. Since the A_M scales inversely with the total city area, the top two schematics are drawn to represent smaller cities.

Supplementary Figure 7: Exponential Distribution Of Thermal Anomalies

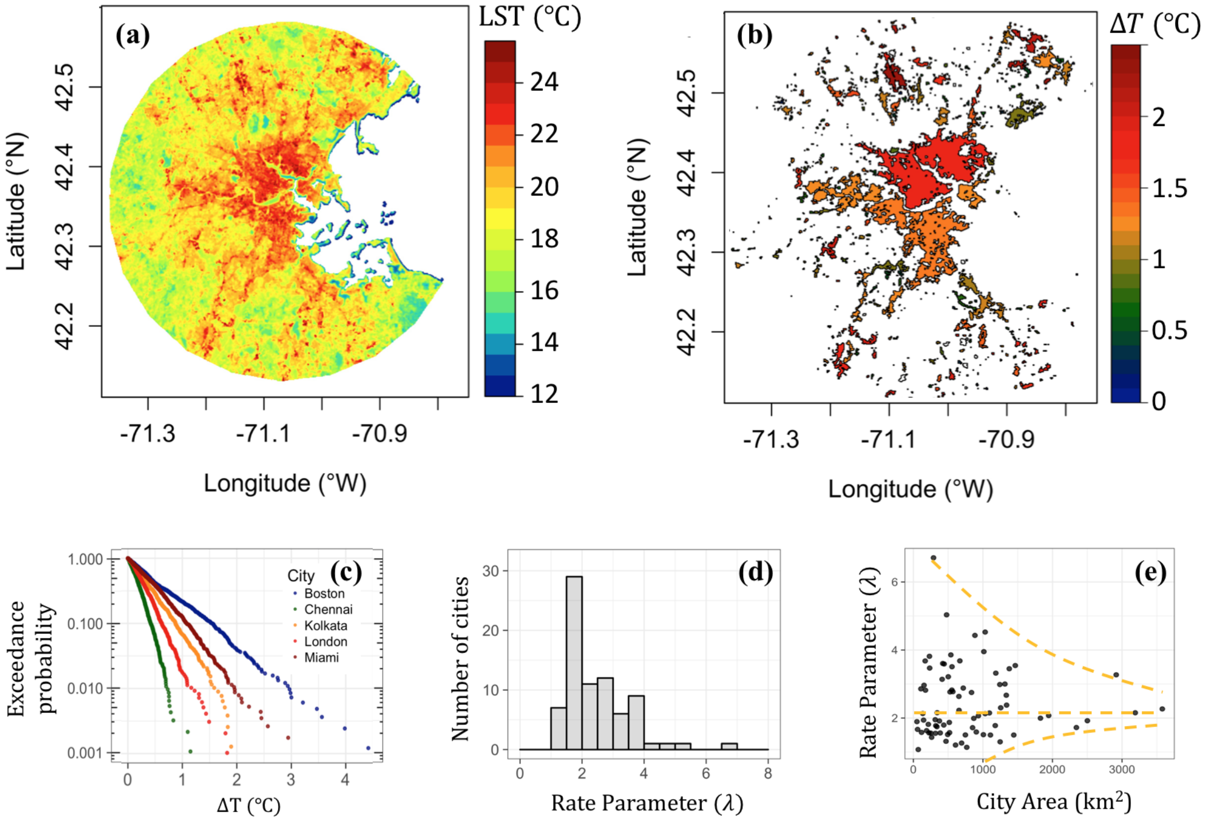

Figure 7: This figure was adapted from ref. [4]. (a) Land Surface Temperature map of Boston (b) Map of heat islets obtained at mode temperature (19°C, in this case) with colour representing the thermal anomaly (ΔT) above the mode. (c) Examples of empirical pdf of ΔT for 5 selected cities shown on a semi-log graph at their respective mode temperatures to illustrate the disparity in exponential pdfs of ΔT. (d) Histogram of rate parameter λ (Eqn. 4) with mean = 2.25. (e) Scatter plot of λ and area of all cities. Yellow dashed lines show the converging behaviour of λ with increasing area.

Supplementary Figure 8: Lacunarity Test

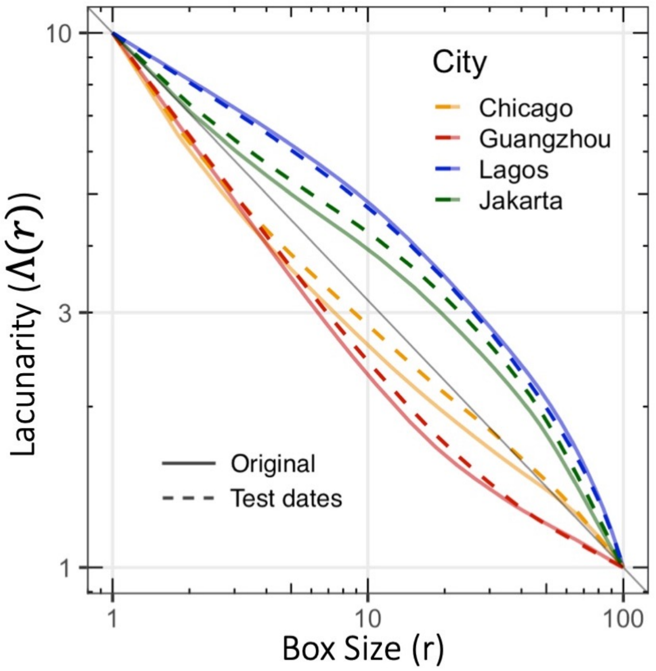

City	Time of acquisition (TOA) of the original image	Original Lacunarity Score	TOA alternate image	New Lacunarity Score
Lagos	(2013-12-18 10:04:25)	0.579	(2016-02-10 10:02:55)	0.684
Jakarta	(2018-07-06 02:59:11)	0.317	(2014-09-13 03:00:14)	0.524
Chicago	(2014-09-23 16:35:10)	-0.313	(2016-05-23 16:34:42)	-0.209
Guangzhou	(2016-02-07 02:52:07)	-0.873	(2017-10-23 02:52:25)	-0.591

Figure 8: This figure serves to illustrate the impact of Landsat imagery acquisition date on our analysis. Lacunarity curves for two separate acquisition dates are plotted and the Lacunarity Score obtained for both dates are presented in the table below. The Lacunarity Score is observed to change, reflecting the change in LST values from one day to the other. However, regardless of the transient nature of temperature, the lacunarity score still reveals the organization (compact vs sprawling) at that instant. We wish to emphasize that our intent is to use Lacunarity as a multi-scale organization indicator. In that sense, the value of the index lies in its slope with respect to the spatial scale (box size), and the aggregated score is calculated to attribute a single value to the curve.

Supplementary Table 1: Koppen Geiger Climate Classification

Group	Letter Code	Type
A: Tropical climates	af	Tropical rainforest climate
	am	Tropical monsoon climate
	aw	Tropical wet and dry climate
B: Dry climates	bsh	Hot semi-arid climate
	bsk	Cold semi-arid climate
	bwh	Hot desert climate
C: Temperate climates	cfa	Humid subtropical
	cfb	Temperate oceanic climate
	csa	Hot-summer Mediterranean climate
	csb	Warm-summer Mediterranean climate
	csc	Cold-summer Mediterranean climate
	cwa	Monsoon influenced humid climate
	cwb	Subtropical highland climate
D: Continental climates	dfa	Hot-summer humid continental climate
	dfb	Warm-summer humid continental climate

Table 1: Koppen-Geiger Climate types [5] [Peel, et al. 2007]. (Source: https://www.pmfias.com/climatic-regions-of-india-stamps-koeppens-classification/)

Supplementary Table 2: List Of Selected Cities, Location, Landsat Scene Information, And Background Koppen Geiger Climate Information Used.

Supplementary Table 3: Results Of Log-Likelihood Statistics For Testing Power Law Tails And Checking Alternate Distributions As Discussed In Text S3

Supplementary Table 4: Metadata On The Final Results Obtained For Final 49 Cities.

NOTE: Tables 2-4 are uploaded as a separate excel file.

References

[1] J. A. Voogt and T. R. Oke, “Thermal remote sensing of urban climates,” *Remote Sensing of Environment*, vol. 86, no. 3, pp. 370–384, 2003.

[2] J. P. Walawender, M. J. Hajto, and P. Iwaniuk, “A new arcgis toolset for automated mapping of land surface temperature with the use of landsat satellite data,” in *Geoscience and Remote Sensing Symposium (IGARSS), 2012 IEEE International*. IEEE, 2012, pp. 4371–4374.

[3] A. Clauset, C. R. Shalizi, and M. E. Newman, “Power-law distributions in empirical data,” *SIAM Review*, vol. 51, no. 4, pp. 661–703, 2009.

[4] A. Shreevastava, P. Rao, and G. McGrath, “Emergent self-similarity and scaling properties of fractal intra-urban heat islets for diverse global cities,” *Physical Reviews E*, 2019.

[5] M. C. Peel, B. L. Finlayson, and T. A. McMahon, “Updated world map of the köppen-geiger climate classification,” *Hydrology and Earth System Sciences*, vol. 4, no. 2, pp. 439–473, 2007.
